# Molecular Epidemiology of Mayaro Virus among Febrile Patients, Roraima State, Brazil, 2018–2021

**DOI:** 10.3201/eid3005.231406

**Published:** 2024-05

**Authors:** Julia Forato, Cássio A. Meira, Ingra M. Claro, Mariene R. Amorim, Gabriela F. de Souza, Stefanie P. Muraro, Daniel A. Toledo-Teixeira, Miguel F. Dias, Cátia A. R. Meneses, Rodrigo N. Angerami, Pritesh Lalwani, Scott C. Weaver, Ester C. Sabino, Nuno R. Faria, William M. de Souza, Fabiana Granja, José Luiz Proenca-Modena

**Affiliations:** Universidade Estadual de Campinas, Campinas, São Paulo, Brazil (J. Forato, M.R. Amorim, D.A. Toledo-Teixeira, R.N. Angerami, F. Granja, J.L. Proenca-Modena);; Laboratório Central de Saúde Pública de Roraima, Boa Vista, Brazil (C.A. Meira, C.A.R. Meneses);; University of São Paulo, São Paulo (I.M. Claro, E.C. Sabino, N.R. Faria);; Imperial College London, London, UK (I.M. Claro, N.R. Faria);; University of Kentucky, Lexington, Kentucky, USA (W.M. de Souza);; Global Virus Network, Baltimore, Maryland, USA (W.M. de Souza, S.C. Weaver);; Federal University of Roraima, Boa Vista (M.F. Dias, F. Granja); Fiocruz Amazônia, Manaus, Brazil (P. Lalwani);; University of Texas Medical Branch, Galveston, Texas, USA (S.C. Weaver);; University of Oxford, Oxford, UK (N.R. Faria)

**Keywords:** Mayaro virus, viruses, arbovirus, mosquito-borne infections, vector-borne infections, febrile illnesses, arthralgia, nanopore sequencing, Amazon, Brazil

## Abstract

We detected Mayaro virus (MAYV) in 3.4% (28/822) of febrile patients tested during 2018–2021 from Roraima State, Brazil. We also isolated MAYV strains and confirmed that these cases were caused by genotype D. Improved surveillance is needed to better determine the burden of MAYV in the Amazon Region.

Mayaro virus (MAYV) is an endemic and neglected mosquitoborne alphavirus that causes acute and chronic debilitating arthritogenic disease in Latin America and the Caribbean (*1*). MAYV infection can cause fever, rash, and arthralgia that can persist for over a year in some patients (*2*). MAYV is transmitted in its enzootic cycle mainly by sylvatic *Haemagogus janthinomys* mosquitoes among nonhuman primates and other mammals, which can lead to spillover to humans (*2*). However, some experimental studies suggest that MAYV could establish a human-amplified cycle in urban environments when transmitted by *Aedes aegypti* and *Ae. albopictus* mosquitoes, which could lead to a larger public health threat (*3*,*4*). No specific antiviral drugs or vaccines are available to treat or prevent MAYV infection.

MAYV infections have been reported in Central and South America since the 1950s (*1*,*2*). However, reports of active circulation of MAYV in human populations remain scarce, even in MAYV-endemic areas. We conducted a molecular epidemiology study to investigate the active circulation of MAYV in patients with acute febrile illness during 2018–2021 from the Amazon Region in Roraima State, Brazil.

## The Study

During December 2018–December 2021, we collected serum samples from 822 patients with acute febrile illness (up to 10 days from onset of symptoms) seeking care at primary health care units across 11 of the 15 municipalities of Roraima State, North Region, Brazil. We collected patient information, such as age, sex, occupation, sample collection data, date of symptom onset, and symptoms, from medical records. We conducted all procedures in accordance with ethics committee approval from the Federal University of Roraima (approval no. 2.881.239) and the University of Campinas (approval no. 5.625.875).

Next, we extracted RNA from all serum samples and performed real-time reverse transcription PCR (rRT-PCR) to detect RNA of MAYV, chikungunya virus (CHIKV), Zika virus, dengue virus (DENV), and Oropouche virus. We also carried out viral isolation in African green monkey kidney cells (Vero CCL-81) with some positive samples. Then, we performed sequencing by using the nanopore approach (*5*) and conducted maximum-likelihood phylogenetic inferences (Appendix).

Of 822 patients tested by rRT-PCR, 190 (23.1%) were positive for >1 arbovirus (Appendix Figure 1). We detected MAYV RNA in 28 (3%) patients, including 15 (54%) patients from Boa Vista, the most populous municipality in Roraima State (Appendix Figure 2). Most (19 [68%]) MAYV cases occurred during January–July 2021. Among patients with rRT-PCR–confirmed MAYV, median age was 31 years (interquartile range 26–43 years), and the male-to-female ratio was 1:5. The most common signs and symptoms reported were fever and myalgia, both of which were reported in 23 (82%) MAYV cases. Arthralgia was reported in 6 (21%) and rash in 3 (11%) cases. The median time between symptom onset and sample collection interval was 3 days (interquartile range 1–4 days). Three (11%) of the MAYV cases were in fishermen who had direct contact with wildlife.

Next, we isolated 2 MAYV strains in Vero CCL-81 cells, and we observed cytopathic effects (CPE) ≈30 hours after inoculation. Then, we performed 3 blind passages and confirmed the viral isolation of 2 strains by using rRT-PCR to detect viral RNA in the supernatant of culture cell passages exhibiting CPE. We observed decreased cycle threshold values representing increased viral loads between passages (Appendix Figure 3). In addition, we confirmed MAYV isolates by using immunofluorescent staining (Appendix Figure 4). Subsequently, we used nanopore sequencing to generate the nearly complete coding sequencing of 3 MAYV strains (2 isolates and 1 directly from a clinical sample). We obtained >90% of MAYV genomes with a mean depth of coverage of >20-folds per nucleotide. We submitted sequences to GenBank (accession nos. PP339762–PP339764).

The maximum-likelihood phylogenetic analysis showed that the MAYV strains circulating in Roraima State in 2021 belong to genotype D (widely dispersed) (Figure). We identified no evidence of recombination in MAYV strains from Roraima State. The novel MAYV strains shared 98.6%–98.9% nucleotide sequence identity with other genotype D strains. The new strains formed a distinct and highly supported monophyletic clade (bootstrap support 100%) and clustered with strains sampled in Peru and Venezuela during 1995–2010.

**Figure:**
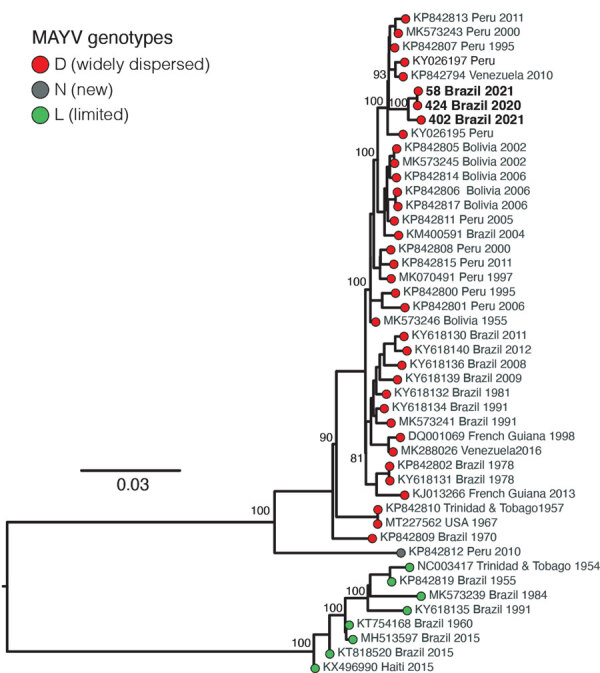
Maximum-likelihood phylogenetic tree of Mayaro virus, Roraima State, Brazil, 2018–2021. Phylogeny is midpoint rooted for clarity of presentation. Bold text indicates 3 new Mayaro virus genomes. Bootstrap values based on 1,000 replicates are shown on principal nodes. Scale bar indicates the evolutionary distance of substitutions per nucleotide site.

Finally, we also detected CHIKV RNA in 16 (2%) and DENV in 146 (17.8%) patients tested (Appendix Figures 1, 5). This number includes 63 patients with DENV serotype 1 and 89 patients with DENV serotype 2. Of those, we identified 6 (1%) cases with co-detection of DENV-1 and DENV-2. We detected most (13 [81%]) chikungunya cases in patients with febrile illness during January–July 2021, overlapping with the peak of detection for MAYV. Conversely, dengue cases were predominantly confirmed (110 [75.3%]) in patients with fever during July 2019–January 2020. All samples tested were negative for RNA of Zika virus, Oropouche viruses, and DENV serotypes 3 and 4.

## Conclusions

This study reports the active MAYV circulation in humans during the concurrent chikungunya and dengue epidemic in Roraima State, Brazil. We found that the MAYV infection cases were caused by genotype D, suggesting that this widespread genotype continued to circulate in the Amazon Region for >60 years. In addition, this same genotype has been detected in outbreaks in Venezuela (*6*,*7*), which, like Guyana, shares borders with Brazil through Roraima State.

Arthralgia has been described as a major clinical characteristic of human MAYV infection (*8*). However, only 21% of MAYV-positive patients reported arthralgia in this study. Our data suggest that laboratory diagnosis of MAYV should be considered for patients with febrile illness in MAYV-endemic areas, even in the absence of clinical characteristics typically associated with MAYV infection. We also found that young adults and men account for most of the MAYV infection cases, probably because of occupational exposure (*9*). Persons who work in forest environments (e.g., in mining, logging, and fishing) could be a bridge to facilitate the eventual introduction and establishment of MAYV transmission in urban settings (*7*). Moreover, the implementation of augmented molecular and genomic surveillance in human and urban vector populations (i.e., *Ae. aegypti* and *Ae. albopictus* mosquitoes) will be critical to monitor the potential establishment of MAYV in a human-amplified transmission cycle.

One limitation of our study is that we focused on active MAYV infections by using a molecular approach; however, further serologic studies are needed to determine the fraction of the population previously infected. Serologic studies can shed light on the potential effect of cross-protection between CHIKV and MAYV in the Amazon Region (*10*). Moreover, the higher percentage (76.9%) of samples negative for the arboviruses tested shows that the metagenomic approach could be useful in further studies to determine the landscape of etiologic agents linked with febrile illness in the triple border region (i.e., Brazil, Guyana, and Venezuela). Further, we were unable to determine whether MAYV infections occurred in urban or forest settings, and we have no follow-up information on MAYV cases.

In conclusion, our study identified the active co-circulation of MAYV, DENV, and CHIKV in patients with febrile illness in Roraima State, Brazil. These findings underscore the critical need for continuous laboratory diagnosis for MAYV to determine the prevalence of MAYV in the Amazon Region and the potential changes associated with urbanization.

Appendix Additional information about molecular epidemiology of Mayaro virus among febrile patients, Roraima State, Brazil, 2018–2021.
