# Supplementary material for: Molecular Epidemiology of Mayaro Virus among Febrile Patients, Roraima State, Brazil, 2018–2021
Source: Emerg Infect Dis. 2024 May;30(5):1013–6. doi: 10.3201/eid3005.231406 (PMC11060474; doi:10.3201/eid3005.231406)
Supplement: Appendix — Additional information about molecular epidemiology of Mayaro virus among febrile patients, Roraima State, Brazil, 2018–2021. [file 23-1406-Techapp-s1.pdf]

Article DOI: https://doi.org/10.3201/eid3005.231406

EID cannot ensure accessibility for Appendix materials supplied by authors. Readers who have difficulty accessing Appendix content should contact the authors for assistance.

Molecular Epidemiology of Mayaro Virus among Febrile Patients, Roraima State, Brazil, 2018–2021

Appendix

Materials and Methods

Blood Samples Collection

Blood samples from patients with acute febrile illness (up to 10 days from onset symptoms) were collected by venipuncture at primary health care units in 11 of the 15 municipalities of Roraima State, Brazil. Next, the samples were sent to the Central Public Health Laboratory to investigate the etiological agent. Lastly, residual serum samples from 822 patients with a volume between 0.2 and 2 mL were provided for this study. All samples were stored at −80°C.

Real-time Quantitative Reverse Transcription-PCR (RT-qPCR) for Mayaro, Chikungunya, Dengue, Zika, and Oropouche Viruses

Viral RNA was extracted from the serum samples using the QIAamp Viral RNA Mini Kit (QIAGEN, USA) following the manufacturer's instructions. Then, RNA extracted was tested by real-time RT-qPCR targeting Mayaro (MAYV) (*1*), chikungunya (CHIKV) (*2*), dengue serotypes 1 to 4 (DENV 1-4) (*3*,*4*), Zika (ZIKV) (*5*), and Oropouche viruses (OROV) (*6*) using the TaqMan RNA to-CT 1-Step Kit (Applied Biosystems, USA) and qPCR BIO Probe 1-Step Go Lo - ROX Kit (PCR Biosystems, UK). Reactions were performed on QuantStudio 3 (Applied Biosystems, USA). The primers and probes used for the viral detection are described (Appendix Table 1).

Virus Isolation

Nine serum samples that tested positive for RNA MAYV via RT-qPCR were inoculated into Vero cells (CCL-81) for viral isolation (Appendix Table 2). We observed viral growth in 2 samples inoculated (≈22%, 2/9). Briefly, Vero cells were plated in six-well plates at a concentration of 1×10^6 cells per ml (1×10^6 cells per well) in Minimum Essential Eagle's Medium (DMEM), supplemented with 10% fetal bovine serum (FBS), and 1% of penicillin of 10,000 units and 10,000 µg/ml streptomycin solution. Subsequently, the serum samples (n=9) were diluted 1:10 in DMEM, treated with 2% penicillin and streptomycin and added to the monolayer. After a one-hour incubation at 37ºC for adsorption, the inoculum was removed, and the monolayer was maintained in DMEM supplemented with 5% FBS. The cells were kept at 37ºC with 5% CO_2 and monitored for 30 hours until the cytopathic effect (CPE) became visible on an optical microscope. Next, the supernatant was collected and subjected to an RT-qPCR assay (*1*) to confirm viral isolation (two samples), indicated by a decrease in the Ct-value.

Mayaro Virus Genome Sequencing and Analysis

MAYV genome sequencing was performed with samples and isolates with Ct values <30 using the Rapid SMART-9N protocol with the MinION platform (Oxford Nanopore Technologies, UK), as previously described (*7*). The generated raw FAST5 files were then basecalled, demultiplexed, and trimmed using Guppy version 9.4.1 (Oxford Nanopore Technologies, UK). The barcoded files were aligned to the MAYV reference genome (GenBank accession no. NC_003417) using minimap2 v.2.17.r941 (*8*) and converted into BAM files using SAMtools (*9*). Medaka_variants were employed for variant calling, followed by medaka_consensus (Oxford Nanopore Technologies, UK) for consensus sequence building. Genome regions with coverage below 20× were represented by the letter "N". NanoStat version 1.6.0, Samtools stats, and samtools depth (*10*) were applied to compute the genome statistics.

Phylogenetic Analysis

The three novel MAYV genomes with >90% coverage were generated and aligned with the non-redundant MAYV strains with complete coding sequences available in the GenBank database as of September 15, 2023. Then, we performed a multiple sequence alignment (MSA) built using MAFFT version 7.450 (*11*), and manual adjustment was conducted using Geneious Prime 2023.0.4. The dataset was screened for recombination events using all available methods in RDP version 4 (*12*), but no evidence of recombination was found. A maximum-likelihood

(ML) phylogeny tree was performed using IQ-TREE version 2 under a GTR + I + γ model determined by ModelFinder (*13*,*14*). The ultrafast-bootstrap approach with 1,000 replicates was used to determine the statistical support for nodes for the ML phylogeny. The phylogenetic tree was visualized using Figtree 1.4.2 (http://tree.bio.ed.ac.uk/software/figtree/).

Immunofluorescence Assay

The immunofluorescence assays were conducted using the MAYV isolate #1 from patient RR-736. Briefly, Vero cells (CCL-81) were cultured in chamber slides at a concentration of 5×10^5 cells per mL (1×10^5 cells per well) in DMEM, supplemented with 10% FBS and 1% of a 1:1 mixture of penicillin (10,000 units/ml) and streptomycin (10,000 μg/mL), and incubated in a 37°C incubator with 5% CO_2 for 24 hours. Subsequently, the cells were infected with the MAYV isolate #1 for 1 hour using a multiplicity of infection (MOI) of 1. For the assay, cells were first blocked with 5% bovine serum albumin (BSA) solution and incubated with BD Cytofix/Cytoperm Fixation and Permeabilization Solution (Becton Dickson, EUA). Afterward, the primary antibodies anti-Mayaro virus E2 antibody, clone MAY-134 (Merk, USA), and anti-Vimentin antibody (ABCAM, USA), both at a dilution of 1:100, were added and incubated for 2 hours at room temperature. Next, cells were incubated with secondary antibodies goat anti-rabbit IgG AF750 (ABCAM, USA) and goat anti-mouse IgG AF594 (Thermo Fisher Scientific, USA), both at a dilution of 1:500, for 1 hour at room temperature protected from light. Hoechst dye was added for 20 minutes at room temperature for nuclear staining, also protected from light. Finally, coverslips were mounted using Fluoromount-G Mounting Medium (Invitrogen, USA), and images were obtained using the Leica TCS SP5 II confocal microscope at a magnification of 20× (Leica, Germany) in the Central Laboratory for High-Performance Life Sciences Technologies at the Universidade Estadual de Campinas, Brazil.

References

1. Waggoner JJ, Rojas A, Mohamed-Hadley A, de Guillén YA, Pinsky BA. Real-time RT-PCR for Mayaro virus detection in plasma and urine. J Clin Virol. 2018;98:1–4. PubMed https://doi.org/10.1016/j.jcv.2017.11.006

2. Lanciotti RS, Kosoy OL, Laven JJ, Panella AJ, Velez JO, Lambert AJ, et al. Chikungunya virus in US travelers returning from India, 2006. Emerg Infect Dis. 2007;13:764–7. PubMed https://doi.org/10.3201/eid1305.070015

3. Callahan JD, Wu SJL, Dion-Schultz A, Mangold BE, Peruski LF, Watts DM, et al. Development and evaluation of serotype- and group-specific fluorogenic reverse transcriptase PCR (TaqMan) assays for dengue virus. J Clin Microbiol. 2001;39:4119–24. PubMed https://doi.org/10.1128/JCM.39.11.4119-4124.2001

4. Johnson BW, Russell BJ, Lanciotti RS. Serotype-specific detection of dengue viruses in a fourplex real-time reverse transcriptase PCR assay. J Clin Microbiol. 2005;43:4977–83. PubMed https://doi.org/10.1128/JCM.43.10.4977-4983.2005

5. Balm MND, Lee CK, Lee HK, Chiu L, Koay ESC, Tang JW. A diagnostic polymerase chain reaction assay for Zika virus. J Med Virol. 2012;84:1501–5. PubMed https://doi.org/10.1002/jmv.23241

6. de Souza Luna LK, Rodrigues AH, Santos RIM, Sesti-Costa R, Criado MF, Martins RB, et al. Oropouche virus is detected in peripheral blood leukocytes from patients. J Med Virol. 2017;89:1108–11. PubMed https://doi.org/10.1002/jmv.24722

7. Claro IM, Ramundo MS, Coletti TM, da Silva CAM, Valenca IN, Candido DS, et al. Rapid viral metagenomics using SMART-9N amplification and nanopore sequencing. Wellcome Open Res. 2023;6:241. PubMed https://doi.org/10.12688/wellcomeopenres.17170.2

8. Li H. Minimap2: pairwise alignment for nucleotide sequences. Bioinformatics. 2018;34:3094–100. PubMed https://doi.org/10.1093/bioinformatics/bty191

9. Li H, Handsaker B, Wysoker A, Fennell T, Ruan J, Homer N, et al.; 1000 Genome Project Data Processing Subgroup. The sequence alignment/map format and SAMtools. Bioinformatics. 2009;25:2078–9. PubMed https://doi.org/10.1093/bioinformatics/btp352

10. De Coster W, D'Hert S, Schultz DT, Cruts M, Van Broeckhoven C. NanoPack: visualizing and processing long-read sequencing data. Bioinformatics. 2018;34:2666–9. PubMed https://doi.org/10.1093/bioinformatics/bty149

11. Katoh K, Standley DM. MAFFT multiple sequence alignment software version 7: improvements in performance and usability. Mol Biol Evol. 2013;30:772–80. PubMed https://doi.org/10.1093/molbev/mst010

12. Martin DP, Varsani A, Roumagnac P, Botha G, Maslamoney S, Schwab T, et al. RDP5: a computer program for analyzing recombination in, and removing signals of recombination from, nucleotide sequence datasets. Virus Evol. 2020;7:a087. PubMed https://doi.org/10.1093/ve/veaa087

13. Nguyen LT, Schmidt HA, von Haeseler A, Minh BQ. IQ-TREE: a fast and effective stochastic algorithm for estimating maximum-likelihood phylogenies. Mol Biol Evol. 2015;32:268–74. PubMed https://doi.org/10.1093/molbev/msu300

14. Kalyaanamoorthy S, Minh BQ, Wong TKF, von Haeseler A, Jermiin LS. ModelFinder: fast model selection for accurate phylogenetic estimates. Nat Methods. 2017;14:587–9. PubMed https://doi.org/10.1038/nmeth.4285

Appendix Table 1. Primers and probes used for the detection of arboviruses in this study

Virus	Sequences (5'→3')	Primers and probes	Target	Genome position	Reference
ZIKV	AARTACACATACCARAACAAAGTGGT	Forward	NS5	9365–9390	Balm et al., 2012 (*5*)
Xx	TCCRCTCCCYCTYTGGTCTTG	Reverse	NS5	9466–9446	
Xx	CTYAGACCAGCTGAAR	Probe		9398–9413	
DENV1	GACACCACACCCTTTGGACAA	Forward	NS5	8586-8606	Callahan et al., 2001 (*3*)
Xx	CACYTGGGCTGTCACCTCCAT	Reverse	NS5	8692–8673	
Xx	AGAGGGTGTTTAAAGAGAAAGTTGACACGCG	Probe		8608–8638	
DENV2	CAGGTTATGGCACTGTCACGAT	Forward	M	1605	Johnson et al., 2005 (*4*)
Xx	CCATCTGCAGCAACACCATCTC	Reverse	M	1583	
Xx	CTCCGAGAACAGGCCTCGACTTCAA	Probe		1008	
DENV3	GGGAAAACCGTCTATCAATA	Forward	C	118–221	Callahan et al., 2001 (*3*)
Xx	CGCCATAACCAATTTCATTGG	Reverse	C	241–221	
Xx	CACAGTTGGCGAAGAGATCTCAAGAGGA	Probe		174–202	
DENV4	TGAAGAGATTCTCAACCGGAC	Forward	C	187-207	Callahan et al., 2001 (*3*)
Xx	AATCCCTGCTGTTGGTGGC	Reverse	C	293–275	
Xx	TCATCACGTTTTTGCGAGTCCTTTCCA	Probe		247–273	
CHIKV	AAAGGGCAAACTCAGCTTCAC	Forward	NSP1	874–894	Lanciotti et al., 2007 (*2*)
Xx	GCCTGGGCTCATCGTTATTC	Reverse	NSP1	961–942	
Xx	CGCTGTGATACAGTGGTTTCGTGTG	Probe		899-923	
MAYV	AAGCTCTTCCTCTGCATTGC	Forward	NSP1	51–70	Waggoner et al., 2018 (*1*)
Xx	TGCTGGAAACGCTCTCTGTA	Reverse1	NSP1	141–160	
Xx	TGCTGGAAATGCTCTTTGTA	Reverse2		141–160	
Xx	GCCGAGAGCCCGTTTTTAAAATCA	Probe		116–140	
OROV	TACCCAGATGCGATCACCAA	Forward	N	356–375	de Souza Luna et al., 2017 (*6*)
Xx	TTGCGTCACCATCATTCCAA	Reverse	N	437–456	
Xx	TGCCTTTGGCTGAGGTAAAGGGCT	Probe		409–433	

C, capsid protein; CHIKV, chikungunya virus; DENV, dengue virus; M, matrix protein; MAYV, Mayaro virus; N, nucleoprotein; NSP1, non-structural protein 1; NS5, non-structural protein 5; OROV, Oropouche virus; ZIKV, Zika virus.

Appendix Table 2. Information of MAYV confirmed cases by RT-PCR

Sample ID	Cycle threshold value	Viral isolation
469	40.00	Not performed
470	39.03	Not performed
472	39.29	Not performed
20	38.82	Failed
21	39.98	Not performed
22	39.71	Not performed
49	39.17	Not performed
58	27.03	Successful (Viral growth)
90	39.70	Not performed
92	40.53	Not performed
120	39.87	Not performed
127	40.43	Not performed
166	39.69	Not performed
240	38.90	Failed
285	39.36	Not performed
310	38.03	Failed
329	39.42	Not performed
336	39.34	Not performed
340	39.26	Not performed
352	39.48	Not performed
360	39.43	Not performed
402	36.82	Failed
430	39.23	Not performed
458	39.40	Not performed
396	37.96	Failed
424	30.35	Successful (Viral growth)
66	31.03	Failed
315	33.76	Failed

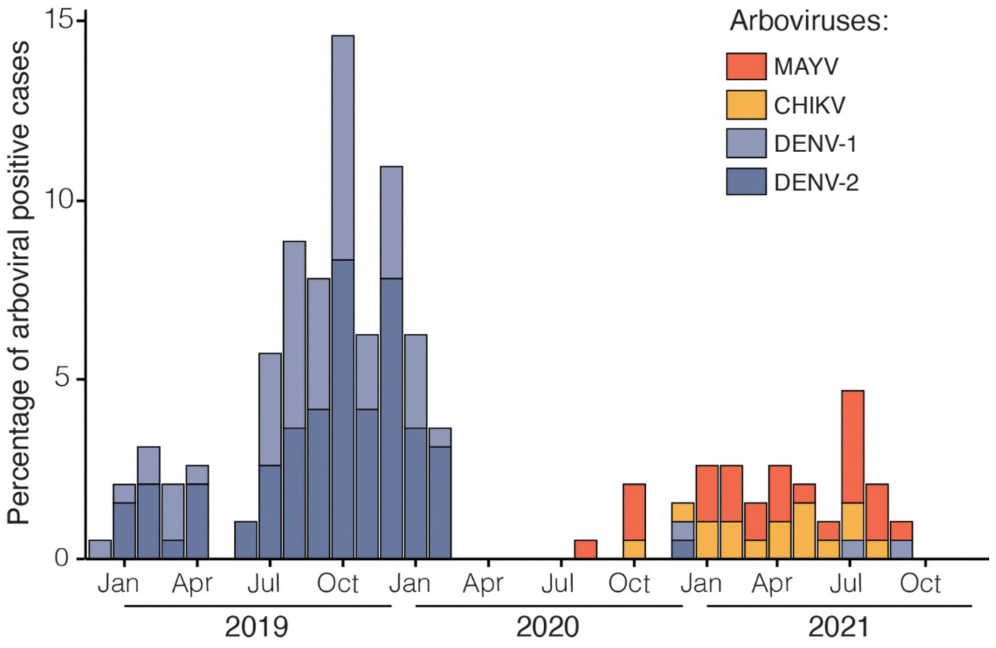

Appendix Figure 1. Molecular epidemiology of Mayaro virus in Roraima State, Brazil. Percentage of PCR-positive cases per fever cases tested for arboviruses (MAYV, CHIKV, DENV-1, and DENV-2) per month in Roraima State, Brazil between 2018 and 2021.

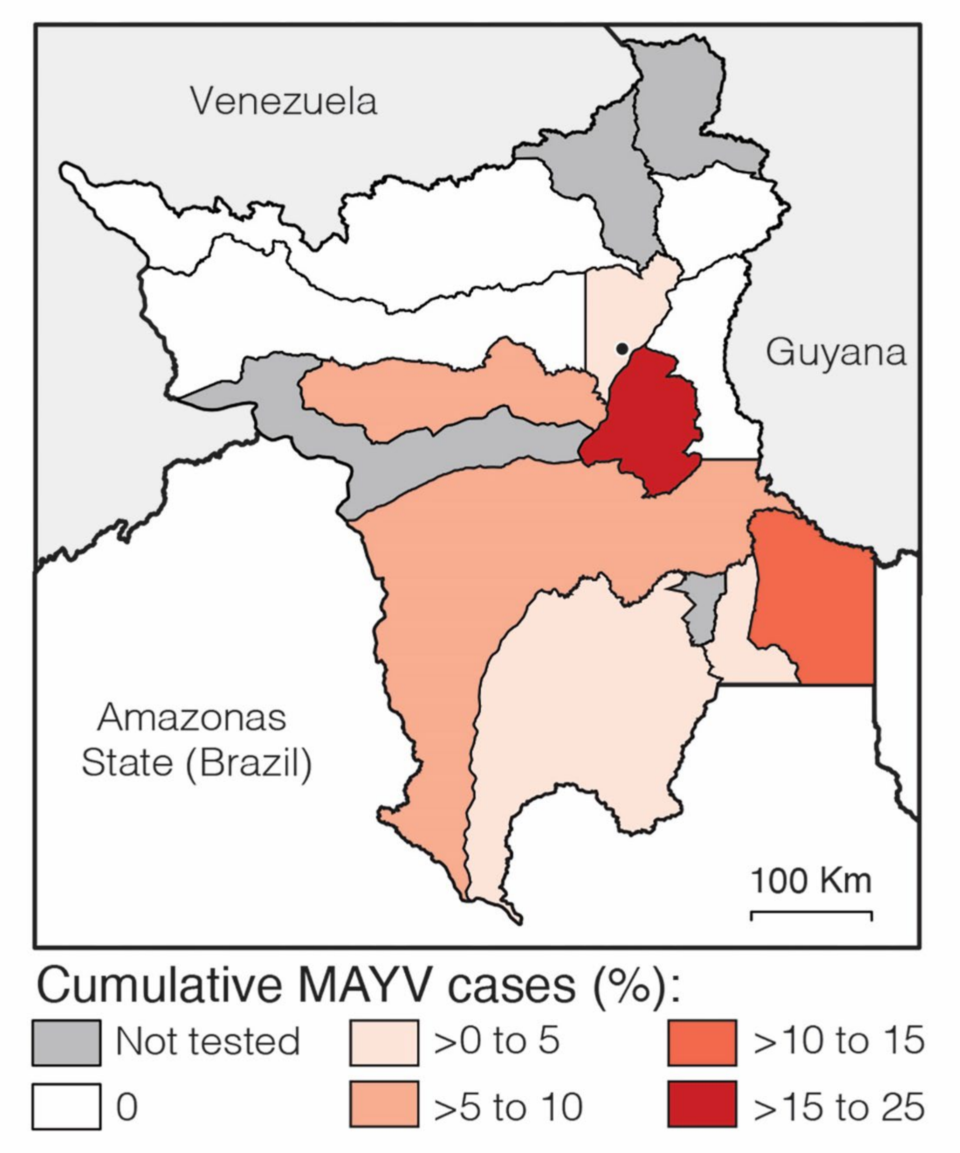

Appendix Figure 2. Molecular epidemiology of Mayaro virus in Roraima State, Brazil. Map of cumulative percentage of MAYV-positive cases per municipality in Roraima State between 2018 and 2021. The frequency of MAYV-positive samples per municipality was: Amajari (0%, 0/3), Alegre Alto (0%, 0/4), Boa Vista (3,9%, 15/384), Bonfim (0%, 0/40), Cantá (0%, 0/4), Caracaraí (9%, 2/23), Caroebe (13%, 1/8), Mucajaí (8%, 1/13), Normandia (0%, 0/5), Rorainópolis (3,5%, 6/172) and São João da Baliza (1,4%, 1/74). No samples from Iracema, Pacaraima, São Luiz, or Uiramutã were tested. Additionally, 92 patients had no information regarding their municipality of residence, including 2 positive samples.

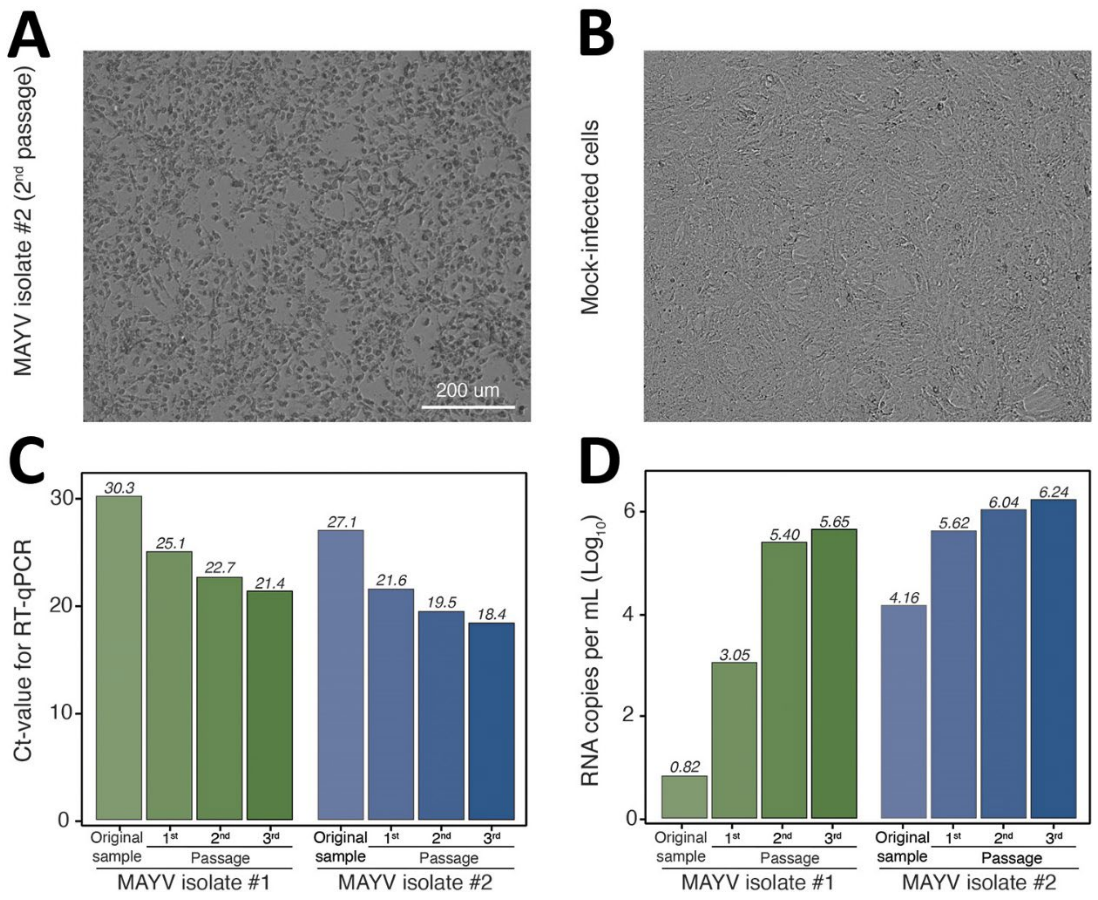

Appendix Figure 3. Mayaro virus isolates in culture cells obtained from serum samples of febrile patients from Boa Vista municipality in 2020 and 2021, Roraima State, Brazil. MAYV isolates were obtained from patient RR-424 (isolate #1) and RR-58 (isolate #2) (A) Vero CCL-81 cells with the cytopathic effect caused by MAYV infection 30 hours post-infection. B) Mock-infected Vero CCL-81 cells (negative control). C). Cycle threshold (Ct)-value obtained by RT-qPCR from MAYV isolates and original samples. D) Estimate MAYV RNA copies per mL obtained by RT-qPCR from MAYV isolates and original samples. MAYV, Mayaro virus.

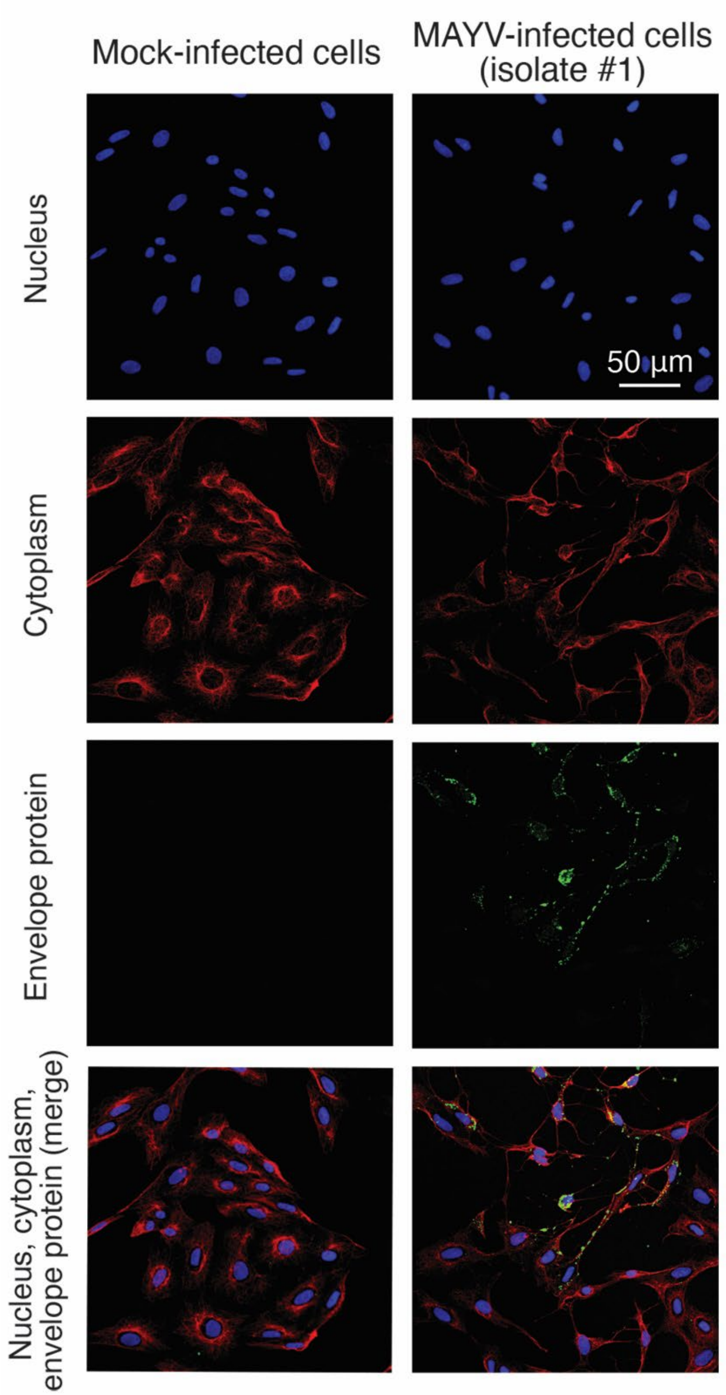

Appendix Figure 4. Immunofluorescence of MAYV isolate. The images show staining of mock-infected Vero CCL81 cells and of Vero CCL81 cells inoculated with MAYV isolate #1 from patient RR-407. Cells were stained with antibodies against vimentin for visualization of the cytoplasm (red fluorescence, row 2), and MAYV envelope 2 protein (green fluorescence, row 3). Nuclei were labeled with Hoechst dye (blue fluorescence, row 1). Slides were analyzed by confocal microscopy, and images were merged with ImageJ (merge, row 4). MAYV, Mayaro virus. DENV, dengue virus. CHIKV, chikungunya virus.

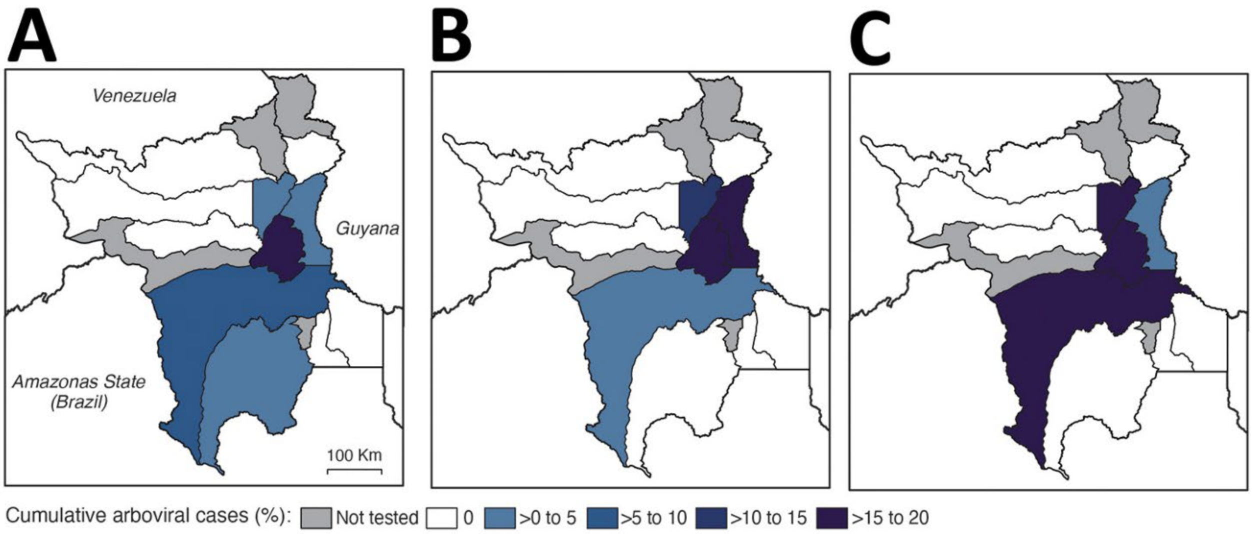

Appendix Figure 5. Maps of distribution of chikungunya and dengue cases in Roraima State, Brazil. Cumulative percentage of positive cases of chikungunya (A), dengue serotype 1 (B), and dengue serotype 2 (C) per municipality in Roraima State between 2018 and 2021. The frequency of CHIKV-positive samples per municipality was: Amajari (0%, 0/3), Alegre Alto (0%, 0/4), Boa Vista (1%, 4/384), Bonfim (5%, 2/40), Cantá (0%, 0/4), Caracaraí (9%, 2/23), Caroebe (0%, 0/8), Mucajaí, (0%, 0/13), Normandia (0%, 0/5), Rorainópolis (5%, 8/172) and São João da Baliza (0%, 0/74). The frequency of DENV1-positive samples per municipality was: Amajari (0%, 0/3), Alegre Alto (0%, 0/4), Boa Vista (13%, 50/384), Bonfim (5%, 2/40), Cantá (25%, 1/4), Caracaraí (17%, 4/23), Caroebe (0%, 0/8), Mucajaí, (0%, 0/13), Normandia (0%, 0/5), Rorainópolis (0%, 0/172) and São João da Baliza (0%, 0/74). The frequency of DENV2-positive samples per municipality was: Amajari (0%, 0/3), Alegre Alto (0%, 0/4), Boa Vista (18%, 70/384), Bonfim (5%, 2/40), Cantá (25%, 1/4), Caracaraí (9%, 2/23), Caroebe (0%, 0/8), Mucajaí, (0%, 0/13), Normandia (0%, 0/5), Rorainópolis (3,5%, 6/172) and São João da Baliza (1%, 1/74). No samples from Iracema, Pacaraima, São Luiz, or Uiramutã were tested. Additionally, 92 patients had no information regarding their municipality of residence, including 4 positive samples to DENV-1 and 12 to DENV-2. Km, kilometers.
